# Chromosomal aberration arises during somatic reprogramming to pluripotent stem cells

**DOI:** 10.1186/s13008-020-00068-z

**Published:** 2020-11-03

**Authors:** Xinyu Liu, Conghui Li, Kang Zheng, Xiaofeng Zhao, Xiaofeng Xu, Aifen Yang, Min Yi, Huaping Tao, Binghua Xie, Mengsheng Qiu, Junlin Yang

**Affiliations:** 1 Key Laboratory of Organ Development and Regeneration of Zhejiang Province, College of Life and Environmental Sciences, Hangzhou Normal University, 311121 Hangzhou, China; 2 College of Life Sciences, Zhejiang University, 310058 Hangzhou, China; 3 Department of Anatomical Sciences and Neurobiology, University of Louisville, 40292 Louisville, KY USA

**Keywords:** Induced pluripotent stem cell, Reprogramme, Karyotype, Chromosomal aberration, Genetic stability

## Abstract

**Background:**

Reprogramming somatic cells to induced pluripotent stem cells (iPSCs) has opened new therapeutic possibilities. However, karyotypic abnormalities detected in iPSCs compromised their utility, especially chromosomal aberrations found at early passages raised serious safety concerns. The mechanism underlying the chromosomal abnormality in early-passage iPSCs is not known.

**Methods:**

Human dermal fibroblasts (HDFs) were stimulated with KMOS (KLF4, cMYC, OCT4 and SOX2) proteins to enhance their proliferative capacity and many vigorous clones were obtained. Clonal reprogramming was carried out by KMOS mRNAs transfection to confirm the ‘chromosomal mutagenicity’ of reprogramming process. Subculturing was performed to examine karyotypic stability of iPSCs after the re-establishment of stemness. And antioxidant N-acetyl-cysteine (NAC) was added to the culture medium for further confirmming the mutagenicity in the first few days of reprogramming.

**Results:**

Chromosomal aberrations were found in a small percentage of newly induced iPS clones by reprogramming transcription factors. Clonal reprogramming ruled out the aberrant chromosomes inherited from rare karyotypically abnormal parental cell subpopulation. More importantly, the antioxidant NAC effectively reduced the occurrence of chromosomal aberrations at the early stage of reprogramming. Once iPS cell lines were established, they restored karyotypic stability in subsequent subculturing.

**Conclusions:**

Our results provided the first line of evidence for the ‘chromosomal mutagenicity’ of reprogramming process.

## Background

Induced pluripotent stem cells (iPSCs) have become a valuable model system for studying tissue development and related human diseases, holding great promise for autogenous cell therapy [1].Further investigation focusing on the genetic stability of iPSCs is necessary, especially as genetic abnormalities in their therapeutic derivatives might harbor the risk of tumorigenesis [2]. Affirming a normal karyotype becomes a basic requirement when cultivating a iPS cell clone, since chromosomal abnormalities are detected from time to time in iPS cell clones by cytogenetic method [3–10]. For late-passage iPS cell clones, it is generally believed that chromosomal aberrations accumulate in the process of culture adaptation in vitro [1, 4, 6]. For early-passage iPS cell clones, the origin of abnormal chromosome has been puzzling. It was reported that chromosomal abnormalities not found in parental cells have been detected in iPSC lines as early as passage 5 (P5) [10], suggesting that the abnormal chromosomes observed in early-passage iPSCs might be derived from the large-scale genetic abnormalities that occurred during reprogramming. However, since they were observed more than one month after the end of reprogramming, it is difficult to rule out the possibility that the aberrations occurred during the cell proliferation stage. Therefore, it is not straightforward to determine the origin of ‘chromosomal mutagenicity’ of reprogramming process. Another possibility is that the genetic aberrations may arise from rare karyotypically abnormal parental cell subpopulation, but no effective method has been established to exclude this possibility [9]. For the above reasons, single karyotypically normal somatic cell-derived clone could be ideally used as appropriate parental cells for reprogramming to prove the ‘mutagenicity’ of reprogramming. However, it is technically challenging or impossible to grow primary somatic cells from single cell to a clone due to their limited life span.

Here, we developed a strategy to increase proliferative capacity of human primary cells by KMOS (KLF4, cMYC, OCT4 and SOX2) protein stimulation, and obtained individual cell-derived karyotypically normal clones. Then, clonal reprogramming was achieved by KMOS mRNAs transfection, and cytogenetic examination detected abnormal karyotypes in a small fraction of iPS cell clones, which demonstrated that reprogramming process itself did trigger chromosomal aberrations. And this find was further confirmed by the performance of antioxidant in reducing karyotypically abnormal iPS cell clones in the first few days of reprogramming.

## Results

### Classification of chromosomal aberrations following somatic reprogramming

**Fig. 1:**
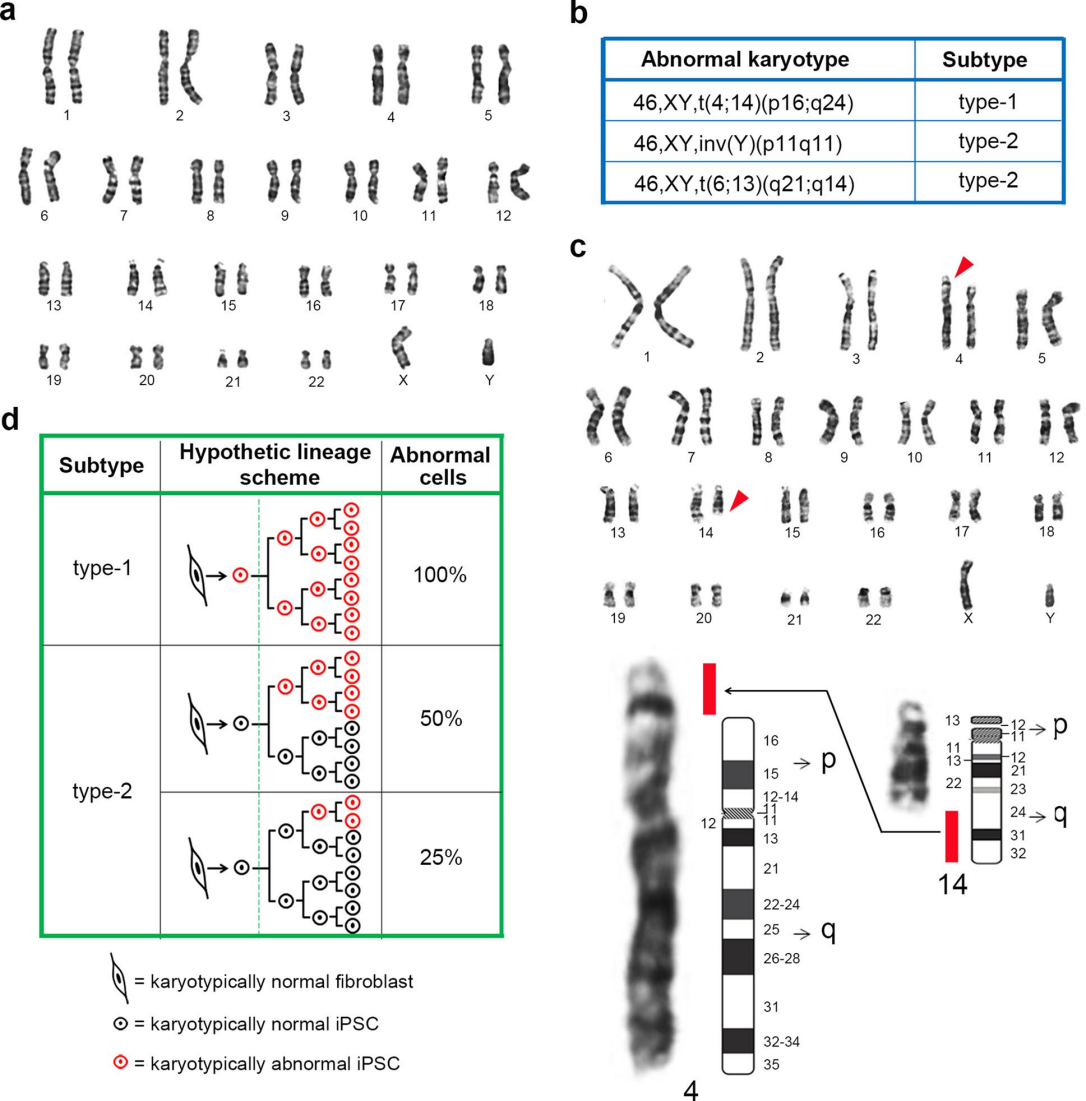
karyotypically abnormal iPS cell clones detected after reprogramming. **a** HDFs were detected to have normal karyotype before reprogramming. **b** List of the abnormal karyotypes detected in new iPS cell clones. **c** Representative abnormal karyotype. A chromosomal fragment on the long arm of chromosome 14 translocated to the short arm of chromosome 4. **d** Hypothetic cell lineages of the appearance of chromosome aberration in type-1 and type-2 karyotypically abnormal iPS cell clones in somatic reprogramming to pluripotent stem cells

Synthetic modified KMOS mRNAs (KLF4, cMYC, OCT4 and SOX2) were transfected into HDFs with normal karyotype (Fig. 1a) to initiate the reprogramming process. Following transfections, human iPSC-like colonies were randomly picked and seeded into 24-well plates at one clone per well (Passage 1). Eight days later, cells were processed for karyotyping. Although the vast majority of detected clones maintained their normal karyotypes, a few of them (3 of the 49 clones, 6.12%) were found to be chromosomally abnormal (Fig. 1b). In one iPS cell clone, the same chromosomal aberration was found in every progeny cell (Fig. 1b, d). This type of aberration is referred to as type-1 clone, and we suspected that the mutation occurred during the reprogramming process (Fig. 1d). The other two karyotypically abnormal iPS clones were found to carry chromosomal aberrations only in partial cells (26.7% and 53.3%, respectively), and they were referred to as type-2 clones (Fig. 1b, d). In these two type-2 clones, all karyotypically abnormal cells also carried the same chromosomal aberration, suggesting that they were derived from one mutated cell (Fig. 1d). Intriguingly, the proportion of chromosomally abnormal cells in these two clones was either one fourth or one half, implying that chromosomal aberrations occurred during the first or second cell division after the fate conversion from somatic cell to iPSC (Fig. 1d).

**Fig. 2:**
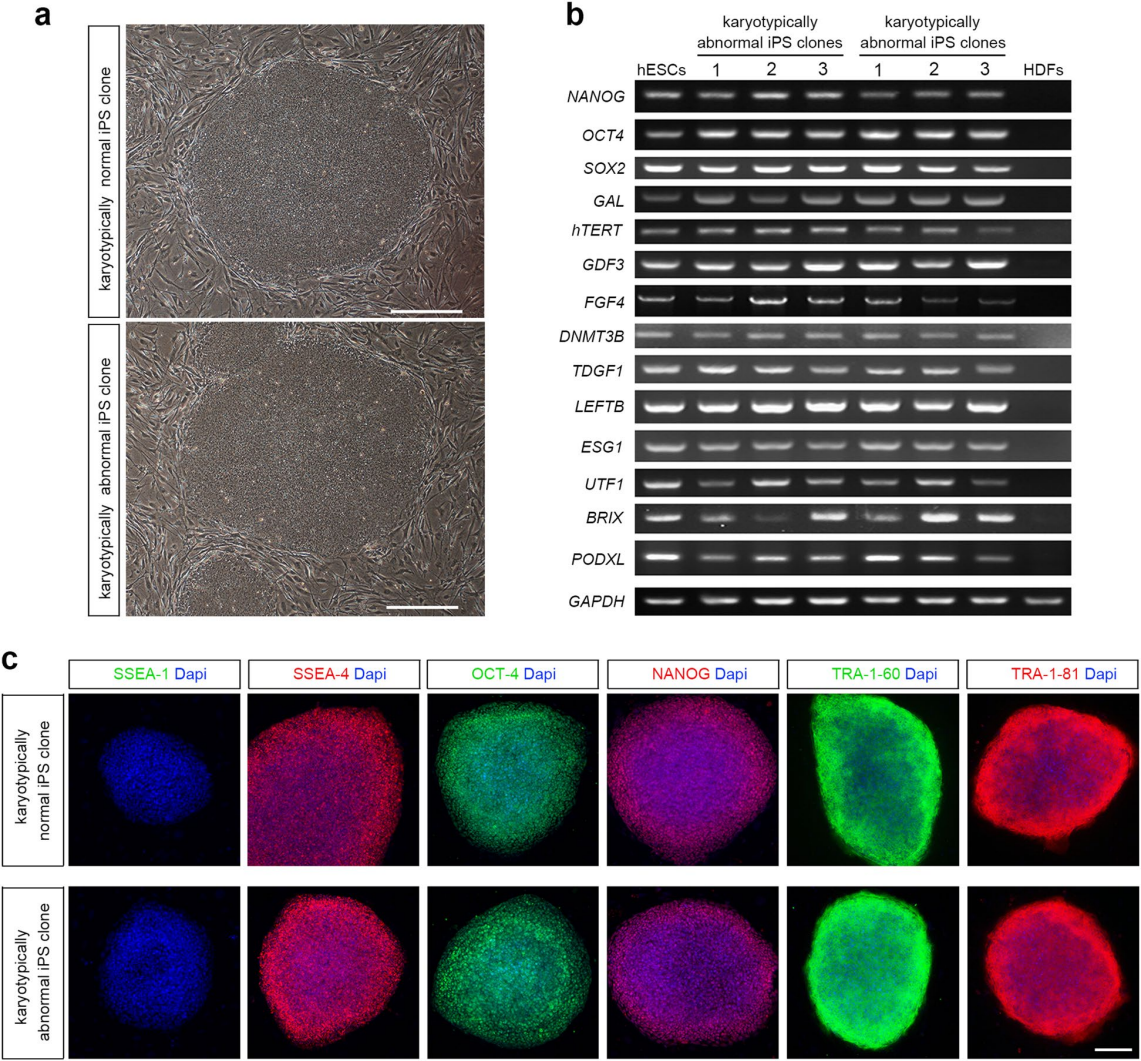
Karyotypically abnormal iPS cell clones showed similar properties to those in normal ones and human embryonic stem cells (hESCs). **a** Morphologies of karyotypically normal and abnormal iPS cell clones. **b** RT-PCR analysis of pluripotent cell-marker genes. **c** The immunophenotype of karyotypically normal and abnormal iPS cell clones. Scale bars, 100 µm

Besides the normal typical morphology (Fig. 2a), RT-PCR showed that the karyotypically abnormal iPS cell clones expressed pluripotent marker genes such as NANOG, OCT4, SOX2 and GAL at similar levels to those in normal ones and human embryonic stem cell (hESC) line H9 (Fig. 2b). The immunophenotype of karyotypically abnormal iPS cell clones were also similar to those of normal ones (Fig. 2c). Furthermore, teratoma were also formed after transplanting karyotypically abnormal iPSCs into immunodeficient nude mice, and three germ-layer tissue cells were detected, showing the pluripotency of their differentiation (Additional file 1: Figure S1). Thus, the chromosomal aberrations did not appear to compromise the differentiation potential of iPSCs. Conceivably, destructive chromosomal aberrations also inevitably occurred in the process of reprogramming, but they were difficult to detect due to their adverse effects on the survival or self-renewal of iPSCs.

## Evidence for mutagenicity during reprogramming process

**Fig. 3:**
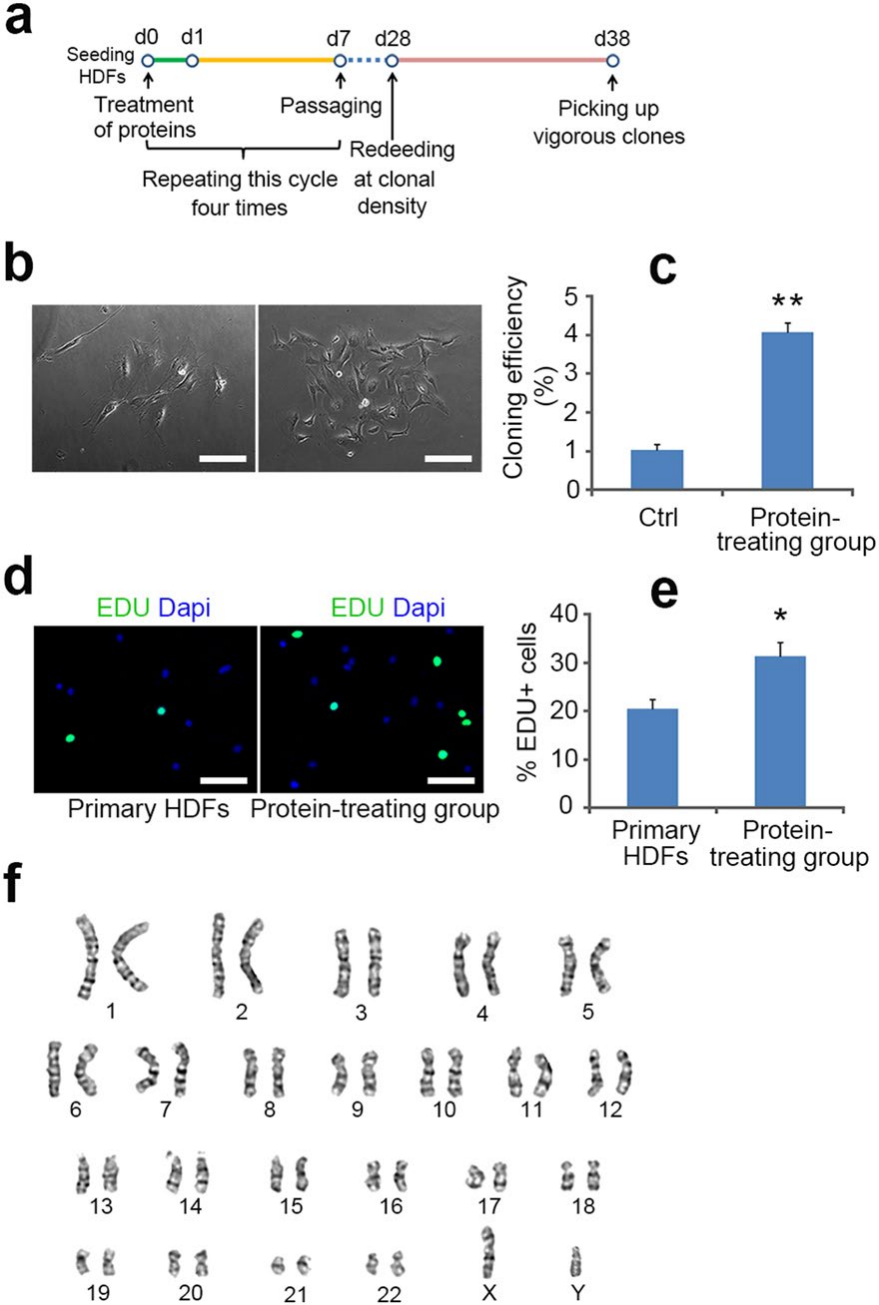
Establishment of single HDF-derived karyotypically normal clones by KMOS protein transduction. **a** The schematic protocol depicts a repeated process and the timeline for generating vigorous HDF clones. **b** Representative image of a HDF clone of control group (left) and a vigorous HDF clone induced by KMOS proteins (right). **c** Quantification of cloning efficiencies in control groups and KMOS protein treatment groups. **d** Representative images of EDU^+^ cells in primary HDF cultures (left) and the cells amplified from vigorous HDF clone (right). EDU (Life Technologies, MA) was added to a final concentration of 10 µM for 24 h. **e** Quantification of EDU^+^ cells in primary HDF cultures and KMOS protein-treating groups. **f** Representative karyotype of vigorous HDF clone, no chromosomal aberration was detected. Statistical analyses are presented as Mean ± SD, n = 3. *P < 0.05, **P < 0.01. Scale bars, 100 µm

Karyotyping only involves the examination of chromosomes at the metaphase stage of mitosis. HDFs isolated from skin tissue were a mixed population, and it is conceivable that some exiguous karyotypical abnormalities may not be detected in the mixture. Therefore, it remains plausible that iPS cell clones with abnormal karyotypes, especially type-1 clones, may be reprogrammed from rare chromosomally abnormal parental cells in this study. To rule out this possibility, we obtained single cell-derived karyotypically normal HDF clones to perform clonal reprogramming. When HDFs were plated at clonal density, only a small percentage (1.02 ± 0.15%) of cells grew into clones (Fig. 3c), and these clones showed poor proliferative capacity with significant aging characteristics (cells became larger with emerging processes) (Fig. 3b). Therefore, it is technically challenging to derive a large number of cells from a single primary clone for somatic cell reprogramming. It was reported that addition of four reprogramming proteins (KLF4, cMYC, OCT4 and SOX2) fused with 9 arginine (9R, a cell-penetrating peptide sequence) to the culture medium can significantly enhance cellular proliferative capacity [11]. When HDFs were incubated with 293T extracts expressing each reprogramming protein, the majority of recombinant reprogramming proteins was translocated into the nucleus, while small amount remained in the cytoplasm (Additional file 1: Figure S3). Therefore, HDFs were tested with the combined cell extracts of four 293T cell lines expressing individual reprogramming factor. After 16 hour of protein transduction, cells were cultured in fibroblast medium for 6 additional days, followed by digesting and passaging (Fig. 3a). After four cycles of repeated protein treatment and subculturing, their cloning ability improved dramatically (Fig. 3c) and many vigorous clones were obtained (Fig. 3b). These clones could be further subcultured for amplification, and EDU labeling showed their significantly higher proliferative capacity compared to primary HDFs (Fig. 3d, e). More importantly, they maintained the typical morphology of fibroblasts and Vimentin expression (Additional file 1: Figure S2), while the immunophenotypes of pluripotent markers such as SSEA-4, OCT4, NANOG, TRA-1-60 and TRA-1-81 were negative (Additional file 1: Figure S2). Furthermore, their karyotypes remained normal and no chromosomal aberration was detected (Fig. 3f).

**Fig. 4:**
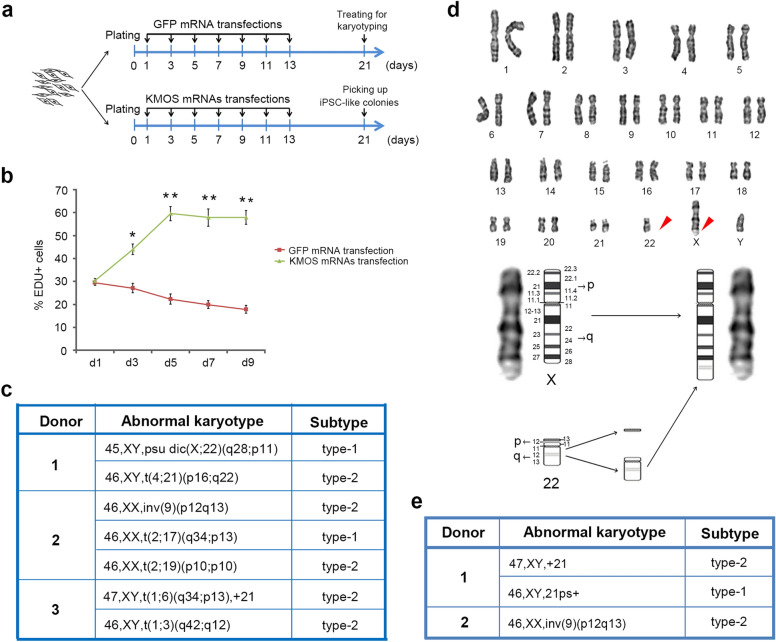
Occurrence of chromosomal aberrations during clonal reprogramming process. **a** The schematic protocols depict two treatments for HDF clones. **b** Quantification of EDU^+^ cells in GFP mRNA transfection and KMOS mRNAs transfection. **c** List of the abnormal karyotypes detected in human dermal fibroblast (HDF) clonal reprogramming. **d** Representative abnormal karyotype detected in HDF clonal reprogramming. Red arrowheads indicate an abnormal chromosome X and a lost chromosome 22. **e** List of the abnormal karyotypes detected in human hair follicle cell (HHFC) clonal reprogramming. Statistical analyses are presented as Mean ± SD, n = 3. *P < 0.05, **P < 0.01

The vigorous HDF clones derived from three donors were randomly selected, and each clone was subjected to two treatments i.e. KMOS mRNAs transfection and GFP mRNA transfection according to the protocol described in Fig. 4a. In KMOS mRNAs transfection group, cellular division rapidly accelerated, and the EDU positive rate on d5 increased to the highest value, which was about twice that of d1(Fig. 4b). While the KMOS mRNAs transfection group obtained a 0.5–0.8% iPSC induction efficiency, no iPSC-like colony was found in the GFP mRNA transfection group. As a result, human iPSC-like colonies were only picked from the KMOS mRNAs group for karyotyping. While the vast majority of clones kept the primary normal karyotype, chromosomal aberrations were found in three donor HDF-derived iPS cell clones (Fig. 4c, d; Additional file 1: Table S1). These results confirmed the ‘chromosomal mutagenicity’ of reprogramming process. The chromosomal aberrations appeared to occur randomly, and no apparent locations or patterns were noticed (Fig. 4c). To exclude the effects of mRNA transfection on karyotypic stability, we examined the karyotypes of cells of GFP mRNA transfection group and found no chromosomal aberration (data not shown). Furthermore, KMOS proteins exhibited similar robust effect on improving the cloning ability of human hair follicle cells (HHFC) (data not shown). Unsurprisingly, we also detected karyotypically abnormal iPS cell clones from the two donor HHFC clonal reprogramming (Fig. 4e; Additional file 1: Table S1). Together, these findings demonstrated that reprogramming process mediated by KMOS mRNA transfection could trigger chromosomal aberration at a low frequency.

## Restoration of karyotypic stability in established iPS cell lines

**Fig. 5:**
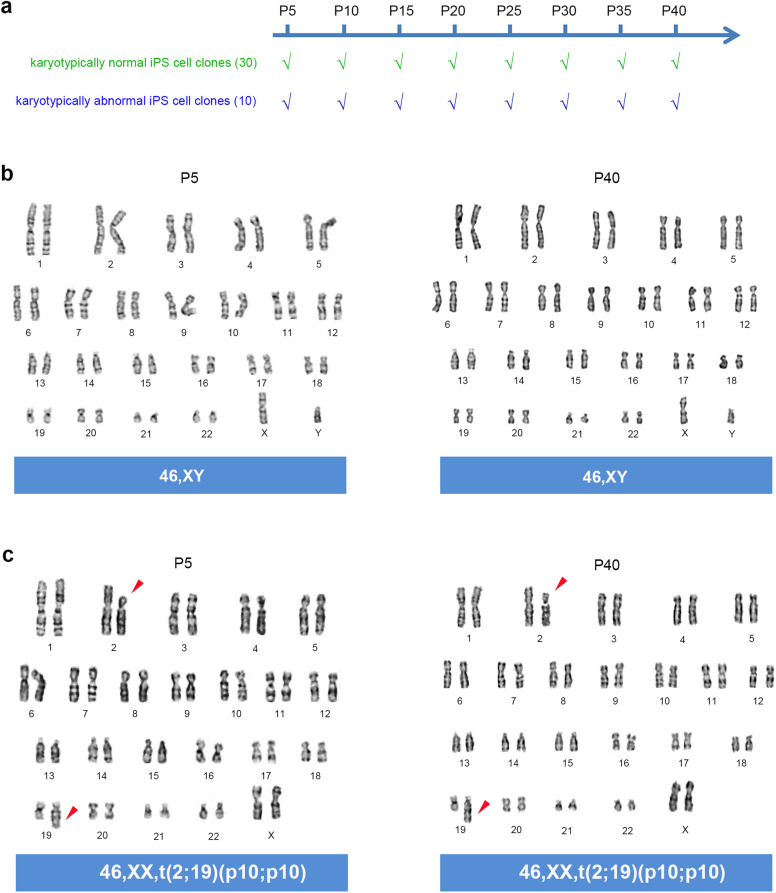
Human iPS cell clones showed karyotypic stability during subculturing. **a** A graph depicts the karyotype stability analysis in established iPS cell lines. ‘√’ indicates that the karyotypes remain unchanged. **b** Representative karyotypes of a karyotypically normal iPS cell clone during subculturing. **c** Representative karyotypes of a karyotypically abnormal iPS cell clone during subculturing. Red arrowheads indicate abnormal chromosome 2 and 19

The emergence of karyotypically abnormal iPS cell clones also prompted us to investigate whether it was caused by the deterioration of chromosomal stability after reprogramming. Therefore, we tracked the karyotypic stability of iPS cell clones in the subsequent subculturing. In this study, 30 karyotypically normal iPS cell clones and 10 abnormal clones were selected and passaged every 6 days by collagenase IV digestion, and karyotyping was performed every 5 generations (Fig. 5a). For karyotypically normal iPS cell clones, their chromosomes kept unchanged (Fig. 5a, b). Of the karyotypically abnormal clones, the primary aberrations were retained, and no new aberrations were observed throughout the subculturing (Fig. 5a, c). These results indicate that iPSCs possess a reliable mechanism for karyotypic stability after the re-establishment of stemness.

## Antioxidants reduced chromosomal aberrations during reprogramming

**Fig. 6:**
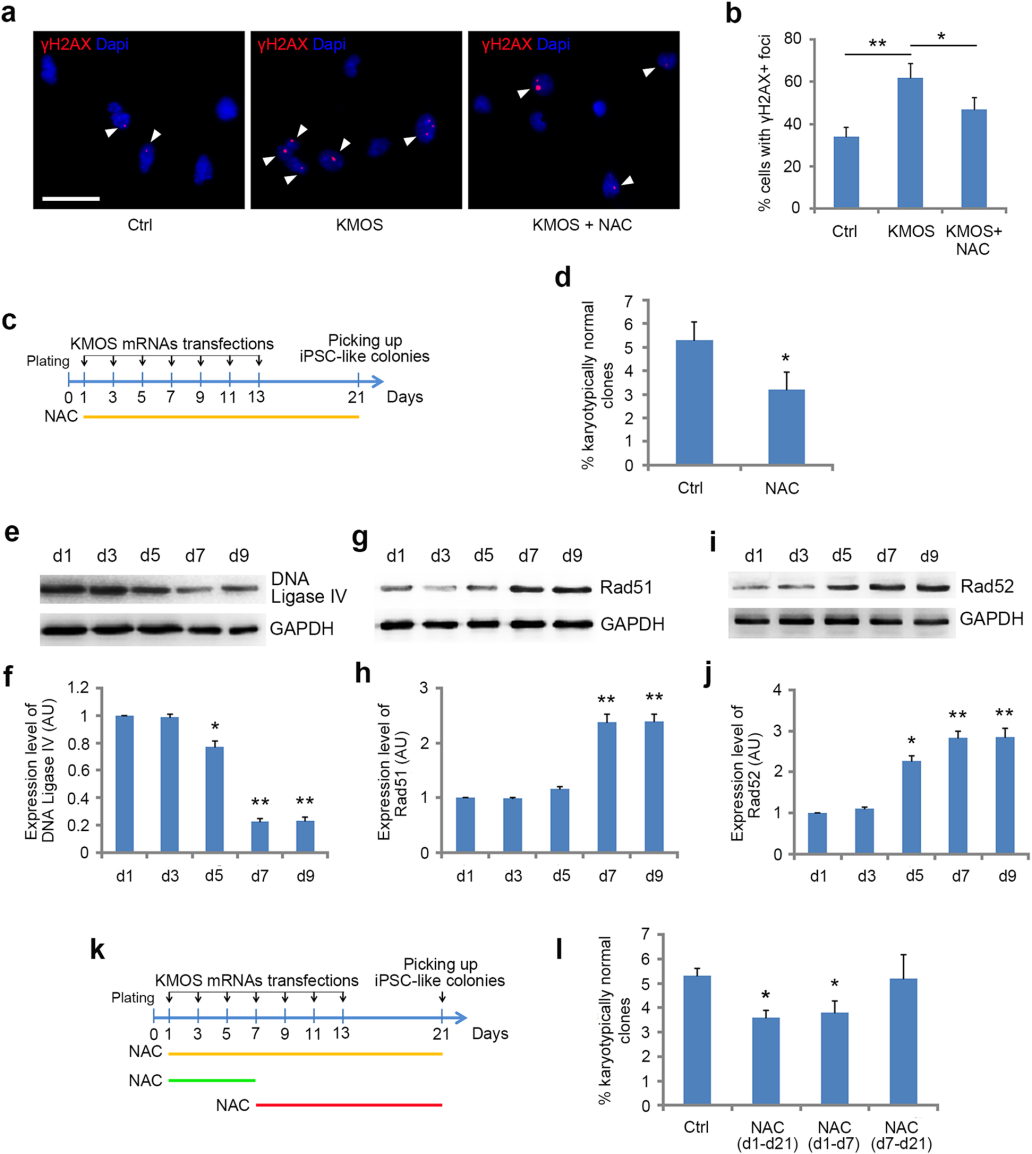
Antioxidants reduced the karyotypically abnormal iPS cell clones. **a**, **b** Immunostaining of γH2AX during reprogramming. Cells at day 4 of transfection with KMOS mRNAs were stained and 100 cells of each condition were counted for γH2AX-positive foci. **c** Schematic diagram of the RNA-based reprogramming regimen with NAC added throughout the reprogramming. **d** The percentage of karyotypically abnormal iPS cell clones in reprogramming cultures treated with NAC or not. **e**, **f** Western blotting and quantitative analysis of DNA Ligase IV in a series of different time points during the reprogramming process. **g**, **h** Western blotting and quantitative analysis of Rad51 in a series of different time points during the reprogramming process. **i**, **j** Western blotting and quantitative analysis of Rad52 in a series of different time points during the reprogramming process. **k** The schematic protocol depicts NAC treatments at different time points during RNA-based reprogramming. **l** Quantification of the percentage of karyotypically abnormal iPS cell clones in reprogramming cultures treated with NAC at different time periods. Statistical analyses are presented as Mean ± SD, n = 3. *P < 0.05, **P < 0.01. Scale bar: 20 µm

As somatic reprogramming to iPSCs undergoes a rapid increase in the rate of cell division (Fig. 4b), it leads to a sharp increase in energy demand and the transformation of oxidative respiration to oxidative glycolysis [12]. The metabolic transformation causes a significant increase in the level of reactive oxygen species (ROS) [13, 14], and high ROS levels can result in oxidative DNA damage [15–20]. Consistent with this principle, we detected a significant increase in the proportion of KMOS mRNAs transfected fibroblasts with γH2AX foci, a widely used marker for monitoring the levels of DNA double-strand breaks (DSBs), while treatment with the antioxidant N-acetyl-cysteine (NAC) reduced significantly γH2AX-positive cells (Fig. 6a, b). We suspect that the DSBs induced by reprogramming may contribute to the chromosomal aberration, and antioxidant may have the potential to reduce the chromosomal aberrations associated with the reprogramming process. To test this idea, we included the antioxidant NAC in the medium during iPSC induction (Fig. 6c), NAC treatment improved the reprogramming efficiency significantly (Additional file 1: Table S4). Random selection of iPSC-like colonies for karyotyping showed that the percentage of karyotypically abnormal iPS cell clones in the NAC treatment group was significantly lower than that of control (Fig. 6d), indicating that NAC treatment effectively reduced the occurrence of chromosomal aberration.

DSBs are the most common form of DNA damage which can be repaired by two different pathways: error-prone nonhomologous end joining (NHEJ) and error-free homologous recombination repair (HRR) [21]. When DSBs are introduced, embryonic stem cells (ESCs) predominantly adopt high fidelity HRR to repair the lesions rather than NHEJ [22–26]. However, when ES cells differentiate into somatic cells, the expression of HRR-related enzymes is down-regulated, whereas the expression of NHEJ-related enzymes, such as DNA Ligase IV, is up-regulated. As a result, DSB repair pathway shifts from HRR to NHEJ [24, 27]. Consistently, the expression level of DNA Ligase IV during reprogramming displayed a significant downregulation after KMOS mRNAs transfection (Fig. 6e, f). In contrast, the expression levels of the HRR pathway-related proteins Rad51 and Rad52 increased gradually, reaching a peak on day 7 (Fig. 6g–j). These data suggest that the NHEJ pathway slowly attenuates during reprogramming while the high-fidelity HRR pathway gradually increases, in an opposite direction of iPSC differentiation [23, 24].

At the early stage of reprogramming, the DSBs induced by the rapid increase of ROS are repaired mainly by NHEJ pathway, which could contribute to the chromosomal aberration in the process of reprogramming. Consistently, our studies revealed that the percentage of karyotypically abnormal clones was reduced significantly by NAC treatment in the first seven days of reprogramming, and the effect was similar to that of the group treated with NAC throughout the reprogramming (Fig. 6k, l). Moreover, adding NAC to the culture medium after the seventh day had no significant effect (Fig. 6k, l). Collectively, these findings strongly suggested the ‘mutagenicity’ of reprogramming process itself.

## Discussion

Karyotypically abnormal human iPS cell clones were frequently reported previously [3–10]. For karyotypically abnormal early-passage iPS cell clones, it was conjectured that the aberration might inherit from rare parental subpopulation [28], but it is difficult to confirm whether such inherited chromosomal aberration has been pre-existing in the donor. Another possibility is that reprogramming process itself is ‘mutagenic’, and that the chromosomal aberrations detected in some early-passage iPS cell clones may occur during reprogramming. In this study, somatic clonal reprogramming confirmed the latter speculation, which was further supported by the effectiveness of NAC in reducing chromosomal aberrations in the first few days of reprogramming.

Because the proportion of karyotypically abnormal iPS cell clones was less than 7% (Fig. 6, Additional file 1: Table S1), and usually only one type of chromosomal aberration was detected within each clone, it seemed that karyotypic abnormality was a low-probability event in the reprogramming process. However, as the self-renewal of pluripotent stem cells (PSCs) is limited in response to genomic insults, differentiation and apoptosis are potential mechanisms by which PSCs with toxic genomic abnormalities are eliminated [29, 30]. Thus, it is conceivable that some chromosomal aberrations which block reprogramming or inhibit the self-renewal of iPSCs do not emerge in iPSC-like colony and thus are not detected. As such, the actual frequency of chromosomal aberrations during reprogramming may be higher than what we observed. Thus, the chromosomal aberrations detected in iPSC-like colonies in this study should be compatible with the self-renewal of iPSCs. Although some genetic aberrations accumulated during subculturing in vitro may not affect the biological characteristics of pluripotential stem cells (PSCs), they could exert adverse effects on their directed differentiation or the function of their derivatives [2, 8, 34].

The emergence of type-1 clones indicates that some chromosomal distortions occur between the beginning and the end of reprogramming, providing the direct evidence for karyotypic abnormalities accompanied with reprogramming. The appearance of type-2 clones suggests that the first few divisions of iPSC after the transition from somatic cells are still susceptible to chromosomal aberration, and the mechanism of maintaining karyotypic stability is still in the process of maturity. Close inspection of the aberration patterns showed that iPSCs tend to acquire abnormal karyotype at both early and late-passages in subculturing in vitro [4, 6, 7, 28], while ESCs appear to encounter aberrations at later passages [4]. It is very likely that the chromosomal aberrations detected in early-passage iPSCs occurred in the reprogramming process. Our study showed iPSCs revealed excellent karyotypic stability in subsequent subcultures, indicating that once reprogramming is completed thoroughly, the signal pathways associated with karyotypic stability is fully restored. Further research on the ‘mutagenicity’ of reprogramming not only helps to understand the molecular process of reprogramming, but also can be explored to improve cellular genomic stability.

The reprogramming of somatic cells to PSCs is not a natural process, and the artificial dedifferentiation acutely changes the original intracellular activities [14]. For instance, reprogramming is accompanied by a sharp increase of ROS levels [13, 14], which in turn triggers more DSBs [13, 31], posing a threat to genetic stability [31]. Our results showed that adding antioxidant to the culture medium significantly reduced chromosomal abnormality in the early stage of the reprogramming process, indicating that high ROS levels contribute to larger chromosomal abnormalities in addition to the previously reported small DNA alterations, such as insertions and deletions (INDELs) and copy number variations (CNVs) [14, 31]. However, the karyotypical mutation during somatic reprogramming could be a complicated process, the induced stress, such as the antagonism between hyper-transcription and hyper-replications [10], may also contribute to the chromosomal aberrations in addition to molecular changes as described above.

Except for chromosomal structural abnormalities, aneuploids are also detected during reprogramming (Additional file 1: Table S2), which is linked to mitotic nondisjunction errors [32]. Moreover, chromosomal structural and numerical aberrations occur simultaneously in a small number of iPS clones (Fig. 4d). Of the abnormal karyotypes detected in this study, 24% are involved in chromosomal numerical aberrations (Additional file 1: Table S2), while loss or gain of whole-chromosome is the predominant form of chromosomal abnormalities for PSCs cultured continuously in vitro [4, 28, 32, 33]. The gained chromosomes, such as trisomies of chromosomes 12 and 17, may carry genes conducive to cell proliferation and anti-apoptosis, giving cells a selective advantage and resulting in the enrichment of aneuloids in culture [9]. Since there is no long-term culture screening, the chromosomal aberrations detected immediately after reprogramming may truly reflect the abnormalities in their types and frequencies and provide a better model system for investigating the mechanisms underlying chromosomal aberrations.

In addition to chromosomal aberrations, small scale aberrations, such as copy number variation (CNV) and point mutations, have also been constantly detected in iPSCs [34]. Chin et al. first reported a few CNVs in each iPSC line, yet none of them was common among those iPSC lines [35]. A larger-scale study using 32 human iPSC lines identified several recurrent CNVs in human iPSCs [36]. On average, a human iPSC line has ~ 10 mutations in the protein-coding regions [37–43]. At least half of the CNVs or point mutations observed in iPSCs were also found in the donor cells [34, 44]. Thus, it was postulated that some CNVs or point mutations in parental cells may exist at undetectably low frequencies, but they become detectable in the iPSC genome during post-cloning amplification. Therefore, somatic clonal reprogramming described in this study should also be a good strategy to ascertain the origin of CNVs or point mutations detected in iPSCs.

## Conclusions

In summary, we utilized KMOS proteins to enhance HDFs proliferation and obtained individual HDF-derived karyotypically normal clones. Then clonal reprogramming by KMOS mRNAs transfection produced abnormal karyotyping in a small fraction of iPS cell clones, which ruled out the aberrant chromosomes inherited from rare karyotypically abnormal parental cell subpopulation. More importantly, we found that antioxidant can reduce the percentage of the karyotypically abnormal iPS cell clones at the early stage of reprogramming. These results provided the first line of evidence for reprogramming can lead to chromosomal aberrations in newly formed iPS cell clones.

## Materials and methods

### Synthesis of mRNAs

Plasmids for generating KLF4, cMYC, OCT4 and SOX2 (abbreviated as KMOS) mRNAs in vitro were obtained from Addgene: pcDNA3.3_KLF4 (catalog # 26,815); pcDNA3.3_OCT4 (catalog # 26,816); pcDNA3.3_SOX2 (catalog # 26,817); pcDNA3.3_c-MYC (catalog # 26,818), and prepared as previously described [45]. RNAs were synthesized and purified as previously described [10]. KMOS mRNA stocks were mixed in 1:1:3:1 ratio to prepare 100 ng/µl (total) combined mRNA reprogramming cocktails.

## RNA-based reprogramming

Human dermal fibroblasts (HDFs) and human hair follicle cells (HHFCs) [46] were isolated from skin biopsies and hair follicles obtained with informed consent and with an approval from The Medical Ethics Review Committees of Hangzhou Normal University Affiliated Hospital. One day before transfection, primary cells were plated onto Geltrex-coated six-well plates (Coring) at 50,000 cells per well in fibroblast medium (Dulbecco’s modified Eagle’s medium (DMEM) supplemented with 10% fetal bovine serum (FBS), 1 × non-essential amino acids solution (NEAA), 1 × GlutaMAX supplement, 1 × Penicillin-Streptomycin (P/S), all from Gibco). The following day the medium was changed to KOSR medium ((DMEM/F12 with GlutaMAX Supplement, 20% KOSR, 1 × MEM NEAA, 55 µM of 2-mercaptoethanol (β-ME), 1 × P/S, all from Gibco) supplemented with 100 ng/ml bFGF (Peprotech), 200 ng/ml B18R (eBioscience), 1 mM VPA (Sigma) and 10 mM Y27632 ROCK inhibitor (BioMol) equilibrated at 5% CO_2_ for 2 hours. The first transfection was performed 2 h after changing the medium. RNA and RNAiMAX Reagent (Invitogen) were first diluted in Opti-MEM (Gibco), the RNA dose was 1000 ng per well (6-well plate) and was diluted 5 ×, and 5 µl of RNAiMAX Reagent per microgram of RNA was diluted 10 ×. After dilution, these components were mixed together and incubated for 20 minutes at room temperature (RT). The transfection mixtures were then dispensed to each well containing cells. Four hours later, the culture supernatant was replaced with KOSR medium supplemented with fresh 100 ng/ml bFGF, 200 ng/ml B18R, 1 mM VPA and 10 mM Y27632. The transfections were performed every 48 h. After three transfections (day 6), cells were digested with TrypLE Select recombinant protease (Invitrogen), and passaged onto gamma-irradiated human fibroblast feeders with a split ratio of 1:6 followed by the other four mRNA transfections. B18R supplementation was discontinued the day after the final transfection and the cells were grown up to day 21 when the iPSC-like colonies were mechanically picked and transferred to MEF-coated 24-well plates with KOSR medium containing 10 ng/ml bFGF and 5 mM Y27632.

## Preparation of protein extracts

For expressing reprogramming proteins, human transcription factors (KLF4, cMYC, OCT4 and SOX2) were fused with 9R and the myc tag [11]. To establish clones stably expressing all 4 factors, 293T cells were transfected with pCMV hOct4-9R-myc, pCMV hSox2-9R-myc, pCMV hKlf4-9R-myc, and pCMV hc-Myc-9R-myc vectors, respectively and were grown in the presence of 500 µg/ml neomycin (G418). After 12 days of screening, G418 resistant clones were picked up and tranferred into 24-well plates for further amplification. Stable 293T clones with high expression of K,M,O or S protein were identified by western blotting analysis. For preparation of cell extracts, cells were washed in 1 × PBS and pelleted by centrifugation at 400×*g* for 5 min at 4 °C followed by suspension in 1 volume of cold cell lysis buffer (100 mM HEPES, pH 8.2, 50 mM NaCl, 5 mM MgCl_2_, 1 mM dithiothreitol, and a cocktail of protease inhibitors (Roche)), and incubated for additional 40 min on ice. Cells were sonicated on ice, followed by centrifugation at 15,000*g* for 15 min at 4 °C to remove the insoluble components. After filtration though a 0.2 µm membrane, the protein extracts from four transfected 293T cell lines were combined at a 1:1:1:1 ratio, diluted to the final concentration of 500 ng/µl, and frozen rapidly.

## Establishment of vigorous HDF clones

Primary HDFs or HHFCs were plated into gelatin-coated 6-well plate. On the following day, cells were treated with combined 293T cell extracts at 100 µg per well for 16 hr. After washing with 1 × PBS, cells were incubated in fibroblast medium for another 6 days with medium being changed every other day. Cells were then digested with TrypLE Select recombinant protease and passaged onto gelatin-coated culture dishes with a split ratio of 1:6. After 4 cycles of protein treatment and subculturing, cells were digested and plated into gelatin-coated dishes at clonal density via fibroblast medium, change the medium every two days. The vigorous clones were mechanically picked and transferred to 24-well plates for amplification.

## Karyotyping

For karyotyping, Colcemid (Gibco) was added to each well, mixed gently and incubated at 37 °C, 5% CO_2_ for 2 h. After washing with 1 × PBS, cells were digested with trypsin. After centrifugation, the supernatants were discarded and cell pellets were re-suspended in 5 ml of 37 °C hypotonic solution (0.075 M KCl), incubated at 37 °C for further 10 min followed by centrifugation. Cell pellets were gently re-suspended in 5 ml cold fixative, and placed on ice for 20 min, and then centrifuged and re-suspended for three times. After the final centrifugation, the cells were suspended in a few drops of cold fixative, 1–2 drops were placed onto wet and clean slides and were left dry at 37 °C for 3 days. After trypsin treatment, the slides were stained with Giemsa solution for 10 min, followed by proper washing and drying. After sealing, first used a low-power lens for a comprehensive inspection, then switched to a high-power lens, observed and took pictures. At least 30 genomes are selected from each sample for analysis. Karyotyping was performed according to ISCN (2016).

### RT-PCR

Total RNA was purified with Trizol reagent (Invitrogen). One microgram of total RNA was used for reverse transcription reaction with Reverse Transcription System (Promega) and Oligo (dT15) Primer, according to the manufacturer’s instructions. Then PCR was performed with ExTaq (Takara, Japan). Primer sequences are shown in Additional file 1: Table S3.

## Teratoma formation

HiPSCs were suspended in KOSR medium containing 10 ng/ml bFGF, SCID mice were anesthetized with diethyl ether and the cell suspension (1 × 10^6^ cells) were injected subcutaneously into the flank of 6-week-old SCID mice. Tumors harvested at 6–10 weeks were fixed in 4% PFA, and embedded in paraffin. Sections were stained with H&E.

## Immunostaining

Immunochemical analysis was carried out as previously described [47]. Antibodies used include anti-SSEA-1 (1:200, FCMAB117P, Merck), anti-SSEA-4 (1:200, MAB4304, Merck), anti-TRA 1–60 (1:200, MAB4360C3, Merck), anti-TRA-1-81 (1:200, MAB4381C3, Merck), anti-NANOG (1:500, MABD24C3, Merck), anti-OCT-4 (1:200, AB3209MI, Millipore), anti-Vimentin (1:500, ab45939, Abcam). The Alexa-488 or Alexa-594 conjugated second ary antibodies were obtained from Molecular Probes (Thermo fisher). The nucleic acid dye 40,6-diamidino-2-phenylindole (DAPI) was obtained from Roche.

## Western immunoblotting

Western blotting was carried out as previously described [48]. Briefly, cells were lysed in sample buffer plus a cocktail of protease inhibitors (Roche). For each sample, 20 mg of protein was used for electrophoresis in SDS-PAGE gel. Primary antibodies were used as follows: anti-rabbit γH2AX (1:200, ab229914, Abcam), anti-DNA Ligase IV (1:800, ab193353, Abcam), anti-Rad51 (1:2000, ab133534, Abcam), and anti-Rad52 (1:1000, ab124971, Abcam). Horseradish peroxidase (HRP)-conjugated secondary antibody (Promaga) was used at 1:2500. Chemiluminescent signals were detected by autoradiography using the ECL System (Amersham, Piscataway, NJ, USA).

### Statistical analysis

All quantitative data are presented as Means ± SD. Statistical significance of the difference was evaluated by Student’s t-test. P-value < 0.05 was considered statistically significant.

## Supplementary information

**Additional file 1: Figure S1.** Hematoxylin and eosin staining for teratoma derived from karyotypically abnormal iPS cell clone. The resulting teratomas contained various types of tissues representing ectodermal, mesodermal and endodermal differentiation. Mesoderm: muscle (a) adipose tissue (b) and cartilage (d); ectoderm: neural tissue (e) and respiratory epithelium (c); endoderm: epidermis (f). Scale bars, 30 μm. **Figure S2.** The immunophenotype of HDFs stimulated by KMOS proteins. Scale bars, 20 μm. **Figure S3.** HDFs were incubated with 293T extracts expressing each reprogramming protein and subjected to immunocytochemistry using myc antibody. Except for some recombinant reprogramming proteins that remained in the cytoplasm, most of them translocated to the nucleus. Scale bars, 40 μm. **Table S1.** Summary of karyotypical mutation rate during clonal reprogramming. **Table S2.** Summary of abnormal karyotypes arised during reprogramming. **Table S3.** RT-PCR Primer sequences. **Table S4.** Quantification of reprogramming efficiency

## Data Availability

The datasets used and/or analyzed during the current study are available from the corresponding authors on reasonable request.

## Abbreviations

iPSCs: Induced pluripotent stem cells
HDFs: Human dermal fibroblasts
NAC: N-acetyl-cysteine
KMOS: KLF4, cMYC, OCT4 and SOX2
EDU: 5-Ethynyl-2’-deoxyuridine
GFP: Green fluorescent protein
ROS: Reactive oxygen species
DSBs: DNA double-strand breaks
NHEJ: Nonhomologous end joining
HRR: Homologous recombination repair
ESCs: Embryonic stem cells
PSCs: Pluripotent stem cells
INDELs: Insertions and deletions
CNVs: Copy number variations
