# Supplementary material for: Chromosomal aberration arises during somatic reprogramming to pluripotent stem cells
Source: Cell Div. 2020 Nov 3;15:12. doi: 10.1186/s13008-020-00068-z (PMC7641821; doi:10.1186/s13008-020-00068-z)
Supplement: Supplementary file 1 — Additional file 1: Figure S1. Hematoxylin and eosin staining for teratoma derived from karyotypically abnormal iPS cell clone. The resulting teratomas contained various types of tissues representing ectodermal, mesodermal and endodermal differentiation. Mesoderm: muscle (a) adipose tissue (b) and cartilage (d); ectoderm: neural tissue (e) and respiratory epithelium (c); endoderm: epidermis (f). Scale bars, 30 μm. Figure S2. The immunophenotype of HDFs stimulated by KMOS proteins. Scale bars, 20 μm. Figure S3. HDFs were incubated with 293T extracts expressing each reprogramming protein and subjected to immunocytochemistry using myc antibody. Except for some recombinant reprogramming proteins that remained in the cytoplasm, most of them translocated to the nucleus. Scale bars, 40 μm. Table S1. Summary of karyotypical mutation rate during clonal reprogramming. Table S2. Summary of abnormal karyotypes arised during reprogramming. Table S3. RT-PCR Primer sequences. Table S4. Quantification of reprogramming efficiency [file 13008_2020_68_MOESM1_ESM.docx]

**Additional file**

**Additional figures**


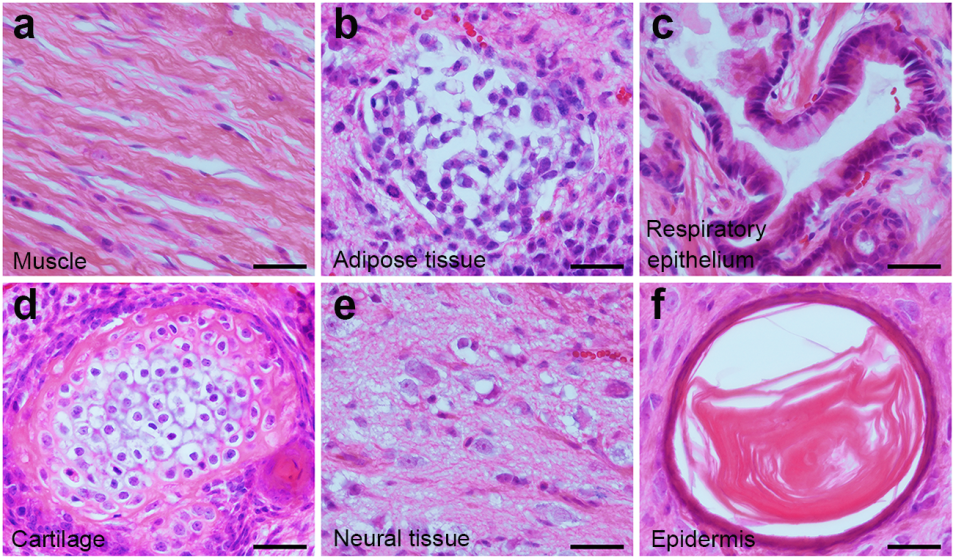


**Fig. S1** Hematoxylin and eosin staining for teratoma derived from karyotypically abnormal iPS cell clone. The resulting teratomas contained various types of tissues representing ectodermal, mesodermal and endodermal differentiation. Mesoderm: muscle (a) adipose tissue (b) and cartilage (d); ectoderm: neural tissue (e) and respiratory epithelium (c); endoderm: epidermis (f). Scale bars, 30 μm.


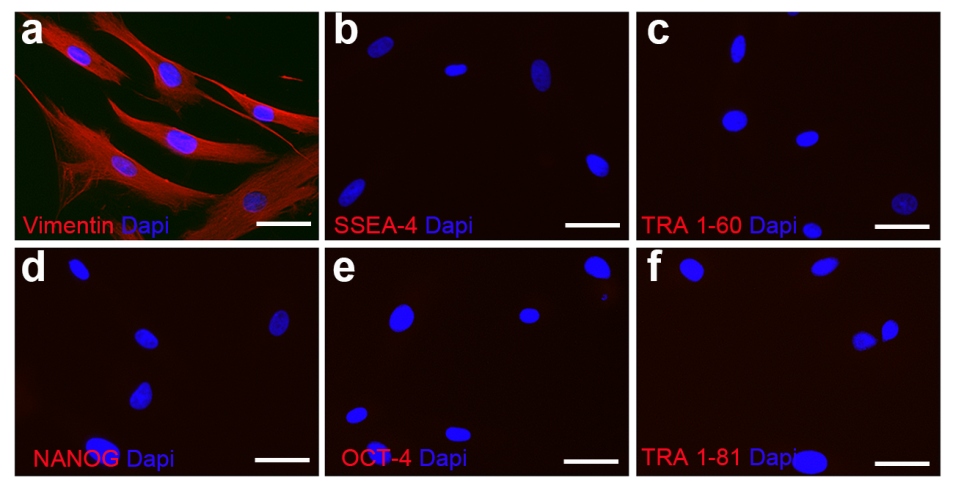


**Fig. S2** The immunophenotype of HDFs stimulated by KMOS proteins. Scale bars, 20 μm.


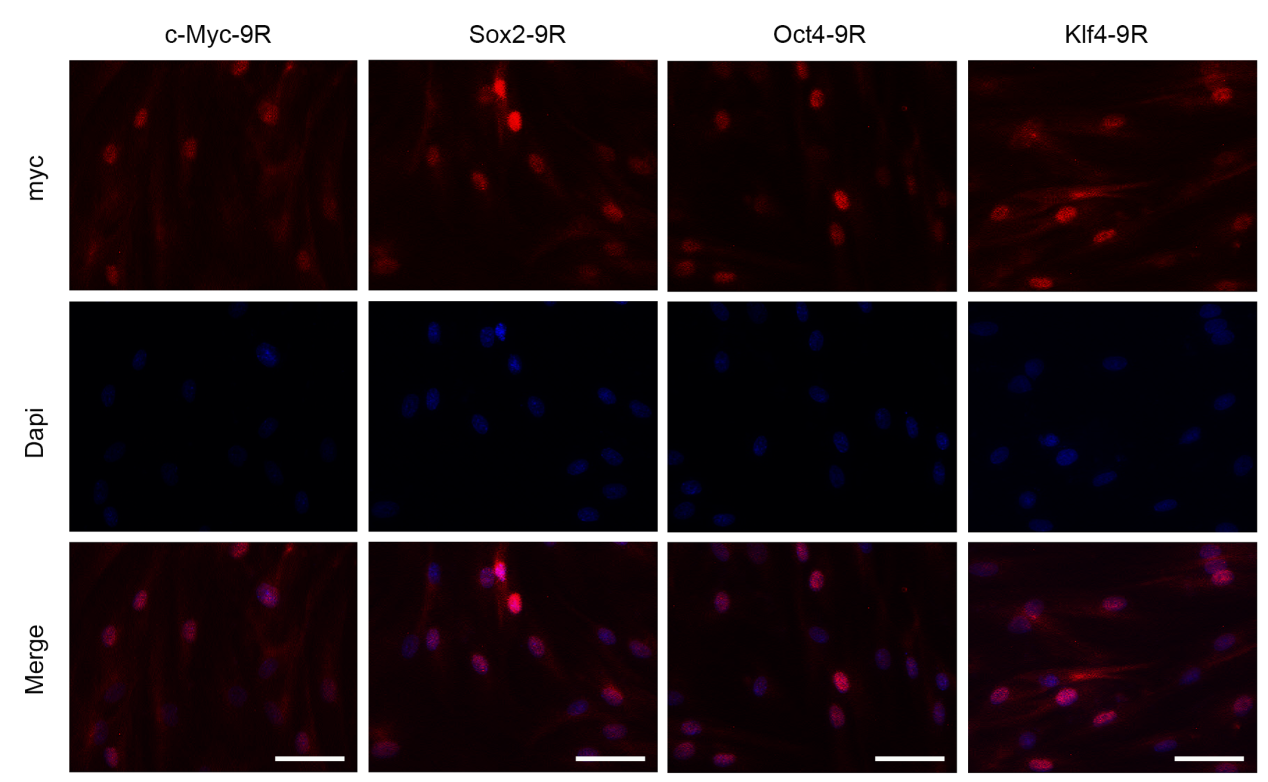


**Fig. S3** HDFs were incubated with 293T extracts expressing each reprogramming protein and subjected to immunocytochemistry using myc antibody. Except for some recombinant reprogramming proteins that remained in the cytoplasm, most of them translocated to the nucleus. Scale bars, 40 μm.

**Additional Table**

**Table S1** Summary of karyotypical mutation rate during clonal reprogramming.

|  | Donor | Total number of iPS clones checked | Karyotypically abnormal iPS clones | Mutation rate (%) |
| --- | --- | --- | --- | --- |
| HDFs | 1 | 41 | 2 | 4.88 |
|  | 2 | 46 | 3 | 6.52 |
|  | 3 | 31 | 2 | 6.45 |
| HHFCs | 1 | 18 | 1 | 5.56 |
|  | 2 | 32 | 2 | 6.25 |

**Table S2** Summary of abnormal karyotypes arised during reprogramming.

|  | **Karyotype** |
| --- | --- |
| Structural aberration | 46,XY,t(1;3)(q42;q12) |
|  | 46,XX,t(X;18)(q23;p11) |
|  | 46,XX,inv(9)(p12;q13) |
|  | 46,XY,inv(9)(p12;q13) |
|  | 46,XX,t(2;17)(q34;p13) |
|  | 46,XYqh+ |
|  | 46,XY,t(5;20)(p15;q12) |
|  | 46,XY,t(8;11)(q12;p13) |
|  | 46,XY,der(14;21)(q10;q10) |
|  | 46,XX,inv(4)(p14q25) |
|  | 46,XY,der(9)t(9;13)(p24;q23)mat |
|  | 46,XY,t(11;22)(q25;q13) |
|  | 46,XX,inv(9)(p12q13) |
|  | 46,XY,der(21;21)(q10;q10) |
|  | 46,XY,t(1;16)(q21;p12) |
|  | 46,XY,t(2;19)(p10;p10) |
|  | 46,XY,t(8;11)(q12;p13) |
|  | 46,XY,t(11;22)(q25;q13) |
|  | 46,XX,t(2;19)(p10;p10) |
|  | 46,XY,inv(4)(p14q27),t(6;13)(q21;q14) |
|  | 46,XX,t(1;8)(p22;p21) |
|  | 46,XY,t(4;14)(p11;q21.2) |
|  | 46,XY,inv(Y)(p11q11) |
|  | 46,XY,t(4;21)(p16;q22) |
|  | 46,XX,del(X)(p11), 16qh+ |
|  | 46,XX,inv(9)(p12q13) |
|  | 46,XY,21ps+ |
| Numerical aberration | 47,XY,+21 |
|  | 47,XXY |
|  | 45,X |
|  | 47,XYY |
|  | 47,Xn,+18 |
|  | 47,Xn,+21 |
| Structural and numerical aberration | 47,XY,t(1;6)(q34;p13),+21 |
|  | 47,XY,inv(9)(p12q13),+21 |
|  | 45,XY,der(14;21)(q10;q10) |
|  | 46,Xn,-16,+mar |
|  | 46,Xn,t(8;11)(q12;p13)pat |
|  | 46,Xn,inv(9)(p12;q13) |
|  | 46,Xn,t(17;20)(p13;q11.2) |
|  | 47,Xn,+der(22)t(11;22)(q25;q13)pat |
|  | 46,Xn,t(2;19)(p10;p10)mat |
|  | 46,Xn,rec(9)inv(9)(p12q13)mat |
|  | 46,Xn,der(6)t(6;9)(q27;p22)mat |
|  | 46,Xn,inv(9)(p12;q21) |
|  | 47,XXX,t(X;6)(q24;p25) |
|  | 45,X,pus dic(X;22)(q28;p11) |

**Table S3** RT-PCR Primer sequences

| Primer | Sequence (5' to 3') |
| --- | --- |
| hNANOG-f | TGA ACC TCA GCT ACA AAC AG |
| hNANOG-r | TGG TGG TAG GAA GAG TAA AG |
| hOCT4-f | GAC AGG GGG AGG GGA GGA GCT AGG |
| hOCT4-r | CTT CCC TCC AAC CAG TTG CCC CAA A |
| hSOX2-f | AGC TAC AGC ATG ATG CAG GA |
| hSOX2-r | GGT CAT GGA GTT GTA CTG CA |
| hGAL-f | GGC CCG AAG ATG ACA TGA AAC C |
| hGAL-r | CCC AGG AGG CTC TCA GGA CCG |
| hTERT-f | TGT GCA CCA ACA TCT ACA AG |
| hTERT-r | GCG TTC TTG GCT TTC AGG AT |
| hGDF3-f | ATG CTA CGT AAA GGA GCT GGG |
| hGDF3-r | GTG CCA ACC CAG GTC CCG GAA |
| hFGF4-f | CAA CGC CTA CGA GTC CTA CA |
| hFGF4-r | GTT GCA CCA GAA AAG TCA GAG |
| hDNMT3B-f | TGC TCA CAG GGC CCG ATA CTT C |
| hDNMT3B-r | TCC TTT CGA GCT CAG TGC ACC ACA A |
| hTDGF1-f | CTG CCT GAA TGG GGG AAC CTG C |
| hTDGF1-r | GCC ACG AGG TGC TCA TCC ATC ACA |
| hLEFTB-f | GGG GAC TAT GGA GCT CAG GGC GAC |
| hLEFTB-r | CAT GGG CAG CGA GTC AGT CTC CG |
| hESG1-f | TCC CGC CGT GGG TGA AAG TTC |
| hESG1-r | ACT CAG CCA TGG ACT GGA GCA |
| hUTF1-f | TCG CTG AAC ACC GCC CTG CTG |
| hUTF1-r | CGC GCT GCC CAG AAT GAA GCC |
| hBRIX-f | CAC GGT ATC ATC CCA AAA GCC AAC C |
| hBRIX-r | ACG CCG ATG CAT GTT TGG TGA CTG G |
| hPODXL-f | AGC CCC ACA GCA GCA TCA ACT ACC |
| hPODXL-r | CCG GGT TGA AGG TGG CTT TGA CTG |
| hGAPDH-f | GAA GGC TGG GGC TCA TTT |
| hGAPDH-r | CAG GAG GCA TTG CTG ATG AT |

**Table S4.** Quantification of Reprogramming Efficiency.

| Experiment | | Cells Plated | Condition | Colonies | Efficiency (%) |
| --- | --- | --- | --- | --- | --- |
| Classification of chromosomal aberrations (HDFs) | | 50,000 | NAC- | 238±33 | 0.48 |
| Clonal reprogramming | HDFs | 20,000 | NAC- | 109±13 | 0.54 |
|  | HHFCs | 20,000 | NAC- | 97±19 | 0.49 |
| Antioxidants (HDFs) | | 50,000 | d1-d21 NAC- | 227±21 | 0.46 |
|  |  |  | d1-d21 NAC+ | 316±27 | 0.63^*^ |
|  |  |  | d1-d7 NAC+ | 339±31 | 0.68^*^ |
|  |  |  | d7-d21 NAC+ | 254±26 | 0.51 |

For each experimental condition, efficiency was calculated by dividing the number of human iPSC-like colonies by the initial number of cells plated. The clonal reprogramming experiment was started in individual wells of a 12-well plate; others in a 6-well plate. Colony counts are shown ± SD, n = 3, ^*^P<0.05.
